# Evaluation of Occupational Exposure Limits for Heat Stress in Outdoor Workers — United States, 2011–2016

**DOI:** 10.15585/mmwr.mm6726a1

**Published:** 2018-07-06

**Authors:** Aaron W. Tustin, Glenn E. Lamson, Brenda L. Jacklitsch, Richard J. Thomas, Sheila B. Arbury, Dawn L. Cannon, Richard G. Gonzales, Michael J. Hodgson

**Affiliations:** ^1^Directorate of Technical Support and Emergency Management, Occupational Safety and Health Administration, Washington, D.C.; ^2^Education and Information Division, National Institute for Occupational Safety and Health, CDC; ^3^Directorate of Enforcement Programs, Occupational Safety and Health Administration, Washington, D.C.

Heat stress, an environmental and occupational hazard, is associated with a spectrum of heat-related illnesses, including heat stroke, which can lead to death. CDC’s National Institute for Occupational Safety and Health (NIOSH) publishes recommended occupational exposure limits for heat stress (*1*). These limits, which are consistent with those of the American Conference of Governmental Industrial Hygienists (ACGIH) (*2*), specify the maximum combination of environmental heat (measured as wet bulb globe temperature [WBGT]) and metabolic heat (i.e., workload) to which workers should be exposed. Exposure limits are lower for workers who are unacclimatized to heat, who wear work clothing that inhibits heat dissipation, and who have predisposing personal risk factors (*1*,*2*). These limits have been validated in experimental settings but not at outdoor worksites. To determine whether the NIOSH and ACGIH exposure limits are protective of workers, CDC retrospectively reviewed 25 outdoor occupational heat-related illnesses (14 fatal and 11 nonfatal) investigated by the Occupational Safety and Health Administration (OSHA) from 2011 to 2016. For each incident, OSHA assessed personal risk factors and estimated WBGT, workload, and acclimatization status. Heat stress exceeded exposure limits in all 14 fatalities and in eight of 11 nonfatal illnesses. An analysis of Heat Index data for the same 25 cases suggests that when WBGT is unavailable, a Heat Index screening threshold of 85°F (29.4°C) could identify potentially hazardous levels of workplace environmental heat. Protective measures should be implemented whenever the exposure limits are exceeded. The comprehensive heat-related illness prevention program should include an acclimatization schedule for newly hired workers and unacclimatized long-term workers (e.g., during early-season heat waves), training for workers and supervisors about symptom recognition and first aid (e.g., aggressive cooling of presumed heat stroke victims before medical professionals arrive), engineering and administrative controls to reduce heat stress, medical surveillance, and provision of fluids and shady areas for rest breaks.

OSHA’s Office of Occupational Medicine and Nursing receives consultation requests from OSHA area offices to address medical questions that arise during OSHA worksite inspections. A master list of these consultations was used to identify 66 heat-related illness consultations during 2011–2016. Three consultations with missing information, 32 indoor incidents, and six that occurred near a heat source were excluded because accurate retrospective heat exposure assessments were not possible. The remaining 25 records were reviewed to assess workers’ personal risk factors, heat acclimatization status, workload, and clothing. Personal risk factors considered in this report were obesity (body mass index ≥30 kg/m^2^), diabetes, hypertension, cardiac disease, and use of certain medications (*1*) and illicit drugs. Workers were considered unacclimatized if they had started a new job within the preceding 2 weeks or if they had recently returned from an absence of >1 week. Workload was classified as light, moderate, heavy, or very heavy, according to ACGIH guidelines (*2*).

Archived climatologic data (i.e., temperature, humidity, wind speed, and sky conditions) were obtained from the nearest National Oceanic and Atmospheric Administration (NOAA) weather station. WBGT at the time of each incident was estimated using a validated heat and mass transfer model (*3*), and Heat Index was computed via a standard NOAA algorithm.* In cases in which the worker’s clothing likely impaired heat dissipation (four), clothing adjustment factors (*2*) were added to the estimated WBGT to determine the effective WBGT (WBGT_eff_). Total heat stress was compared with the applicable NIOSH exposure limit (i.e., the Recommended Exposure Limit for acclimatized healthy workers or the Recommended Alert Limit for workers who were unacclimatized or had personal risk factors). The sensitivity of the exposure limits was defined as the percentage of cases where heat stress met or exceeded the applicable limit.

The sample consisted of 25 heat-related illnesses that occurred during outdoor work, 14 (56.0%) of which were fatal (Table 1). Approximately half (12 of 25) of workers had at least one predisposing personal risk factor. Workload was moderate, heavy, or very heavy in 13 of 14 fatalities; the remaining fatality involved light workload in an unacclimatized worker. Estimated WBGT_eff_ and Heat Index did not differ significantly across categories of workload or acclimatization status (Table 2). The range of WBGT_eff_ was 79°F–94°F (26.1°C–34.4°C). The sensitivity of the NIOSH exposure limits was 100% (14 of 14) for detection of fatal heat stress and 72.7% (eight of 11) for detection of conditions that caused nonfatal illness.

**TABLE 1:** Worker demographic information and job characteristics for 25 outdoor occupational heat-related illnesses — United States, 2011–2016

Characteristic	Fatal illnesses (n = 14)	Nonfatal illnesses (n = 11)	Total sample (n = 25)
Age in years, median (range)	46 (23–64)	17 (15–53)	**36 (15–64)**
Male, no. (%)	14 (100.0)	5 (45.5)	**19 (76.0)**
Unacclimatized to heat, no. (%)	11 (78.6)	1 (9.1)	**12 (48.0)**
Known presence of at least one predisposing personal risk factor, no. (%)*	9 (64.3)	3 (27.3)	**12 (48.0)**
**Estimated workload, no. (%)**			
Light	1 (7.1)	2 (18.2)	**3 (12.0)**
Moderate	5 (35.7)	3 (27.3)	**8 (32.0)**
Heavy	7 (50.0)	6 (54.5)	**13 (52.0)**
Very heavy	1 (7.1)	0 (0.0)	**1 (4.0)**
Work clothing impeded heat dissipation, no. (%)	2 (14.3)	2 (18.2)	**4 (16.0)**

* Obesity, diabetes, hypertension, cardiac disease, and use of certain medications or illicit drugs.

**TABLE 2:** Summary of 25 outdoor heat-related illnesses that were analyzed to evaluate heat stress occupational exposure limits — United States, 2011–2016.

Case no.	Fatality	Acclimatized to heat	Personal risk factor(s)*	Workload level	Clothing adjustment factor	Effective WBGT^†^	Heat Index	Total heat stress above the occupational exposure limit
1	No	Yes	No	Light	None	84°F (29°C)	93°F (34°C)	No
2	Yes	No	Yes	Light	None	86°F (30°C)	92°F (33°C)	Yes
3	No	Yes	Yes	Light	None	90°F (32°C)	103°F (39°C)	Yes
4	No	Yes	No	Moderate	None	79°F (26°C)	85°F (29°C)	No
5	Yes	No	Yes	Moderate	None	80°F (26°C)	86°F (30°C)	Yes
6	No	Yes	No	Moderate	None	81°F (27°C)	90°F (32°C)	No
7	No	Yes	No	Moderate	None	83°F (28°C)	87°F (31°C)	Yes
8	Yes	No	Yes	Moderate	None	85°F (29°C)	90°F (32°C)	Yes
9	Yes	No	Unknown	Moderate	None	86°F (30°C)	96°F (36°C)	Yes
10	Yes	No	Yes	Moderate	+5.4°F (+3°C)	89°F (32°C)	90°F (32°C)	Yes
11	Yes	No	Yes	Moderate	None	93°F (34°C)	104°F (40°C)	Yes
12	No	Yes	Yes	Heavy	None	79°F (26°C)	87°F (31°C)	Yes
13	Yes	No	Yes	Heavy	None	80°F (27°C)	86°F (30°C)	Yes
14	Yes	No	Unknown	Heavy	None	80°F (27°C)	86°F (30°C)	Yes
15	Yes	No	Yes	Heavy	None	83°F (28°C)	97°F (36°C)	Yes
16	No	Yes	No	Heavy	+5.4°F (+3°C)	84°F (29°C)	83°F (28°C)	Yes
17	No	No	Unknown	Heavy	None	85°F (29°C)	91°F (33°C)	Yes
18	No	Yes	Unknown	Heavy	None	85°F (29°C)	92°F (33°C)	Yes
19	No	Yes	Yes	Heavy	None	86°F (30°C)	94°F (34°C)	Yes
20	Yes	Yes	Yes	Heavy	None	90°F (32°C)	110°F (43°C)	Yes
21	No	Yes	No	Heavy	+5.4°F (+3°C)	91°F (33°C)	90°F (32°C)	Yes
22	Yes	No	Yes	Heavy	None	91°F (33°C)	110°F (43°C)	Yes
23	Yes	Yes	Unknown	Heavy	None	92°F (33°C)	106°F (41°C)	Yes
24	Yes	Yes	Unknown	Heavy	+19.8°F (+11°C)	94°F (35°C)	86°F (30°C)	Yes
25	Yes	No	No	Very heavy	None	87°F (30°C)	95°F (35°C)	Yes

**Abbreviation:** WBGT = wet bulb globe temperature.

* Obesity, diabetes, hypertension, cardiac disease, and use of certain medications or illicit drugs.

^†^ Effective WBGT equals measured WBGT plus any applicable clothing adjustment factor.

The median Heat Index was 91°F (33.3°C) and ranged from 83°F to 110°F (28.3°C to 43.3°C). The Heat Index was <91°F (32.8°C) in 12 of 25 cases, including six of 14 fatalities. Among workers wearing a single layer of normal clothing (21), the minimum Heat Index was 85°F (29.4°C), and four of nine nonfatal illnesses and four of 12 fatalities occurred when the Heat Index was between 85°F (29.4°C) and 90°F (32.2°C).

## Discussion

Because WBGT incorporates four environmental factors (air temperature, relative humidity, wind speed, and radiation [often sunlight]) that contribute to heat stress, it is the recommended workplace environmental heat metric. In 2016, NIOSH reiterated this recommendation in an updated publication that defines WBGT-based occupational exposure limits (*1*). The limits were derived from human experiments and have high sensitivity for detecting unsustainable heat stress in laboratory settings (*4*). However, few data have documented the effectiveness of the exposure limits in real-life situations (*1*). The current report partially fills this data gap. In this analysis, the exposure limits had 100% sensitivity for identifying fatal levels of heat stress in outdoor industries. This result suggests that the recommended limits are sufficiently protective of most workers.

Heat Index is an “apparent” temperature that combines humidity and air temperature to quantify what the conditions “feel like” to the human body. Heat Index was designed for the general public, based on algorithms that assume a person is wearing light clothing and walking in a shaded area with a light breeze (*5*). Heat Index does not account for the effects of direct sunlight, stagnant air, work clothing, and strenuous activities. Employers often obtain Heat Index information from publicly broadcasted weather reports or forecasts that do not necessarily reflect conditions at their worksites. These limitations preclude Heat Index from supplanting WBGT as the occupational gold standard. Nonetheless, at outdoor worksites where WBGT is unavailable, Heat Index is sometimes used to estimate environmental heat. This study demonstrates that workers wearing normal clothing are at risk for heat-related illness when Heat Index is ≥85°F (29.4°C). Whenever the Heat Index is ≥85°F, employers should exercise extra vigilance and implement additional precautions (Box), which could include a more accurate WBGT-based environmental heat assessment.

BOX **Protective measures to prevent occupational heat-related illnesses**

- Train supervisors and workers about heat-related signs, symptoms, and first aid.
- Designate someone to monitor heat conditions and oversee protective measures.
- Provide extra protection for new workers until their bodies acclimatize to heat.
- Schedule frequent breaks in a cooler location (e.g., shade or air conditioning).
- Use validated tools, such as CDC’s National Institute for Occupational Safety and Health exposure limits, to assess workplace heat stress.
- Adjust schedules and workload to stay below established heat stress limits.
- Recognize that lower heat stress limits are needed for new workers, those with predisposing conditions, those who perform heavy physical activity, and those who wear hot clothing.
- Provide water or electrolyte-containing beverages.
- Comply with applicable state workplace heat regulations.

Current occupational Heat Index guidance might not be sufficiently protective. For example, although OSHA does not have an enforceable permissible exposure limit for heat stress, OSHA guidance states that a Heat Index of <91°F (32.8°C) is associated with “lower” risk of heat-related illness unless other factors (e.g., direct sun, little air movement, strenuous workload, or nonbreathable clothing) are present (*6*). However, six of 14 deaths in this report occurred when the Heat Index was <91°F. Additional evidence supports the possibility of serious illness when the Heat Index is <91°F. Fourteen percent of moderate to severe heat-related illnesses at a U.S. military training installation (*7*) and at least 25% of heat-related illnesses in Washington agriculture and forestry workers (*8*) occurred when the Heat Index was <90°F (32.2°C). Some employer reports of heat-related hospitalizations to OSHA’s Severe Injury Reports database (*9*) have been associated with a Heat Index of <80°F (26.7°C). A recent mathematical analysis demonstrated that the NIOSH exposure limits can be exceeded when the Heat Index exceeds 85°F (29.4°C) (*10*).

The findings in this report are subject to at least four limitations. First, some workers’ acclimatization status, workload, or clothing might have been misclassified. For example, all workers with >2 weeks of job tenure were considered acclimatized, but during early-season heat waves, some long-term workers might have been unacclimatized to heat. Second, local environmental heat at worksites might have differed from meteorologic data obtained from the nearest NOAA weather station. Third, the WBGT estimation algorithm was subject to small (<1°C) random errors (*3*) and, in some cases, to uncertainties because of reliance on cloud cover as a surrogate for solar radiation measurements. Finally, there was an inability, possibly attributable to the study’s sample size, to detect differences in environmental heat between groups stratified by workload or acclimatization status. Future research could expand upon the findings in this report to define Heat Index-based occupational exposure limits that account for physical activity and acclimatization.

As part of a comprehensive program to prevent heat-related illnesses, employers should measure heat stress throughout the workday, preferably by using WBGT, and take actions to prevent exposure limits from being exceeded. When WBGT is unavailable, a Heat Index threshold of 85°F (29.4°C) could be used to screen for hazardous workplace environmental heat. The comprehensive heat-related illness prevention program should also include an acclimatization schedule for newly hired workers and unacclimatized long-term workers (e.g., during early-season heat waves), training for workers and supervisors about symptom recognition and first aid (e.g., aggressive cooling of presumed heat stroke victims before medical professionals arrive), engineering and administrative controls to reduce heat stress, medical surveillance, and provision of fluids and shady areas for rest breaks (*1*).

**Summary**

**What is already known about this topic?**

Recommended heat stress occupational exposure limits are based primarily on wet bulb globe temperature (WBGT), workload, and acclimatization status. These limits have not been validated at outdoor worksites.

**What is added by this report?**

Among 25 outdoor occupational heat-related illnesses, WBGT-based occupational exposure limits were exceeded for all 14 fatalities and for eight of 11 nonfatal illnesses. Six fatalities occurred when the Heat Index was ˂91°F (32.8°C).

**What are the implications for public health practice?**

Whenever heat stress exceeds occupational exposure limits, workers should be protected by acclimatization programs, training about symptom recognition and first aid, and provision of rest breaks, shade, and water. A Heat Index of 85°F (29.4°C) could be used as a screening threshold to prevent heat-related illness.

## Footnotes

* http://www.wpc.ncep.noaa.gov/html/heatindex_equation.shtml.
